# Matched Filters, Mate Choice and the Evolution of Sexually Selected Traits

**DOI:** 10.1371/journal.pone.0003005

**Published:** 2008-08-20

**Authors:** Konstantinos Kostarakos, Manfred Hartbauer, Heiner Römer

**Affiliations:** Zoology, Karl-Franzens-University, Graz, Austria; University of Exeter, United Kingdom

## Abstract

**Background:**

Fundamental for understanding the evolution of communication systems is both the variation in a signal and how this affects the behavior of receivers, as well as variation in preference functions of receivers, and how this affects the variability of the signal. However, individual differences in female preference functions and their proximate causation have rarely been studied.

**Methodology/Principal Findings:**

Calling songs of male field crickets represent secondary sexual characters and are subject to sexual selection by female choice. Following predictions from the “matched filter hypothesis” we studied the tuning of an identified interneuron in a field cricket, known for its function in phonotaxis, and correlated this with the preference of the same females in two-choice trials. Females vary in their neuronal frequency tuning, which strongly predicts the preference in a choice situation between two songs differing in carrier frequency. A second “matched filter” exists in directional hearing, where reliable cues for sound localization occur only in a narrow frequency range. There is a strong correlation between the directional tuning and the behavioural preference in no-choice tests. This second “matched filter” also varies widely in females, and surprisingly, differs on average by 400 Hz from the neuronal frequency tuning.

**Conclusions/Significance:**

Our findings on the mismatch of the two “matched filters” would suggest that the difference in these two filters appears to be caused by their evolutionary history, and the different trade-offs which exist between sound emission, transmission and detection, as well as directional hearing under specific ecological settings. The mismatched filter situation may ultimately explain the maintenance of considerable variation in the carrier frequency of the male signal despite stabilizing selection.

## Introduction

Many acoustic communication systems involve the production and transmission of specific sound frequencies. The “matched filter hypothesis” [1], [2] argues that receivers should gain an advantage from being selectively tuned to these frequencies, in particular in noisy environments, since the match between the sensitivity of their auditory system and the energy spectrum of the sendeŕs vocalisation would maximize the signal-to-noise-ratio for reception. Experimental evidence for this hypothesis stems from a comparison of basilar papilla tuning in two populations of cricket frogs, with the population in the acoustically more challenging habitat having evolved a filter that better eliminates noise [3], [4]. A match between spectral characteristics of the male call to the tuning of the female hearing organ may also be important by fascilitating intraspecific mate recognition, and could at the same time bias a femalés choice towards particular males ([5] for review). Highly tuned receivers, however, are a challenge for senders, since any deviation from the best frequency in tuning would render their signal less stimulating–and thus less attractive–for receivers. For the evolution of such a communication system one would expect strong stabilizing selection for exactly this best frequency in receiver tuning.

Male field crickets produce almost pure tone calling songs which represent secondary sexual traits subject to sexual selection by female choice [5]. Much attention has been paid in recent years to variation in these male signals within and across populations, and how call parameters such as carrier frequency, call rate or other temporal properties may convey reliable information on male quality [6]–[10]. The carrier frequency (CF) appears of particular interest, since it may be a reliable indicator of male size and reflect the past history of a male [7], [11]. Indeed, there exists intraspecific variation in CF, and female *Gryllus campestris* prefer low frequency calls indicative of large males [12]. A proximate explanation for the choice of females was also offered, based on the tuning of ears around 4 kHz [12]. However, this explanation has never been tested experimentally, and individual differences in female preference functions have rarely been studied (but see e.g. [13] for grasshoppers).

Field crickets are ideal candidates for studying the proximate basis of female preferences because (i) the calling song is almost a pure tone and varies within a population [7]; [14]; [15], (ii) the importance of carrier frequency in eliciting female phonotaxis [7], [16]–[18], (iii) the ears of the females as well as interneurons of the auditory pathway are tuned to the CF of the calling song [12], and (iv) the known function of identified interneurons for positive phonotaxis [19], [20]. The phonotaxis of female crickets towards the male signal appears to be governed by the simple rule “turn to the side stimulated more strongly” (if the temporal patterns at both sides are equal [19]–[23]). By determining both the behavioral preference in standardized choice paradigms, and the frequency tuning of the interneuron relevant for this choice in the same individuals we tested the matched filter hypothesis, and how it might affect female choice and hence selection on the male signal.

In addition to the task of identifying a male of the correct species, a female receiver also needs to localize the sound source. Different from humans or larger mammals, who can exploit interaural time differences and interaural intensity differences (IIDs), the latter caused by diffraction of sound around the head, crickets or other small animals cannot rely on these mechanisms when using lower frequencies with larger wavelength [24]. Instead, the necessary IIDs result from a pressure gradient receiver with a functional three-input system for the sound, provided by a complicated anatomical arrangement of connecting trachea between the ears in the forelegs, and a phase delay mechanism [16; 24]–[27]. A drawback of this mechanism is, however, that directionality is sharply tuned, so that reasonable IIDs for the localization of a calling male are only provided for a narrow range of frequencies. Thus in the receiver, there exists a second, biophysically based “matched filter” depending on sound frequency, with a BF that may or may not be identical to the BF in the tuning of the AN1-neuron, and which strongly affects the receivers̀ ability to localize its mate. Here we show, by examining both “matched filters”, i.e. the frequency tuning filter and the directionality filter in the same individuals, that the frequency providing strongest stimulation for the auditory system may provide only poor directional cues, and that the trade-off between both filters in receivers may drive stabilizing selection of the CF in the male signal.

## Results

### Tuning of receivers and female choice for calling songs differing in CF

**Figure 1:**
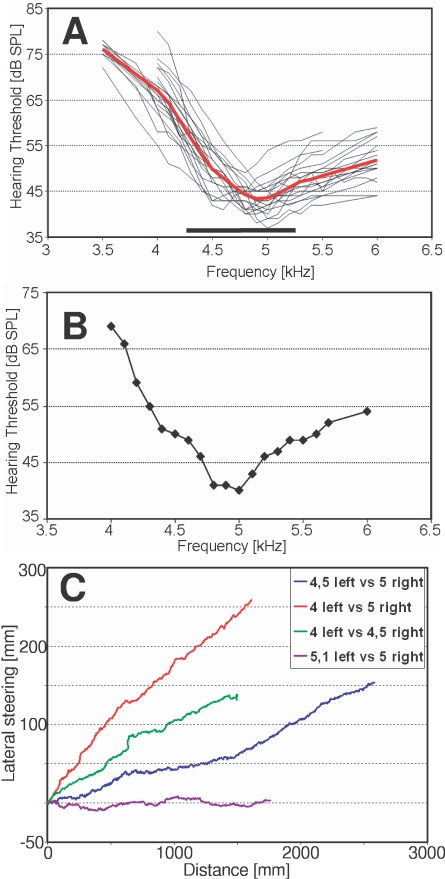
Tuning of AN1-interneuron and its relevance for the preference of females in two-choice tests. (A): Variance of tuning curves (red = average tuning; N = 20) in relation to the range of variation of carrier frequencies of male calls (black bar). (B): Tuning of AN1 in a single female and (C) the degree of lateral steering in a two-choice situation towards a calling song at a CF providing the stronger stimulation (right speaker).

The frequency tuning curve connects measurements of lowest thresholds for various frequencies, and thus identifies frequencies of highest sensitivity (BF). The tuning curve of the AN1-neuron was established in 20 female *G. bimaculatus* between 3.5 to 6 kHz (figure 1A). Females vary with respect to the best frequency and the bandwidth of tuning (mean BF 4.9 kHz, range 4.5–5.2 kHz; mean Q_5_-dB value 660 Hz, range 300–1100 Hz). Starting at the best frequency, the tuning curve shows a strong increase in hearing threshold, with a higher roll-off to lower frequencies compared to higher frequencies. Both the steepness, as well as the BF of AN1 tuning should have a strong influence for the choice of females between signals varying in CF. In a choice between two males, one calling at the BF of the female receiver, the other at a higher or lower CF, the female should consistently prefer the first one, since the signal provides a stimulation which is more intense relative to the alternative.

Figures 1B, C demonstrate, for one individual female, that the predictive value of the AN1-tuning for the preference is very high. AN1 in this female is tuned to 5 kHz, and in a choice with a carrier of 4 kHz the latter signal is perceived as being 29dB less intense. As a result, the female shows strong lateral steering towards the 5 kHz signal. A choice between 4.5 and 5 kHz would result in a smaller difference of 10 dB in favour of 5 kHz, with the female moving with a less strong steering into this direction. Finally, if the alternative signal is at a CF of 5.1 kHz, threshold differences for these two carriers are almost not existent, and the female shows no preference with a net movement into a direction right between both sound sources.

Next, we analysed for each choice pair of CFs whether there was a significant preference for the calling song providing the stronger stimulation. This was based on the respective threshold difference for a given pair of CFs in the AN1 tuning curve, and does not take into account the strength of preference (table 1). In all choice situations which resulted in a net intensity difference of more than 3.4 dB, there was a significant steering towards the signal providing stronger stimulation.

**Table 1:** Summary of results of two-choice-trials between song models differing in CF, in relation to the mean threshold difference of the AN1-neuron for the two involved frequencies, established in each individual female (preferred CF in bold; “z-test”).

Choice for alternative Frequency [kHz]	Intensity difference [dB]	Significance (N)
3.5 versus **5**	32.6	P<0.001 (17)
3.5 versus **5.5**	27.7	P<0.001 (13)
3.5 versus **4.5**	26.2	P<0.001 (18)
4 versus **5**	23.5	P<0.001 (39)
**5.5** versus 4	18.6	P<0.001 (32)
4 versus **4.5**	17.1	P<0.001 (40)
**6** versus 4	15.2	P<0.001 (12)
3.5 versus **4**	9	P<0.001 (15)
6 versus **5**	8.3	P<0.001 (18)
4.5 versus **5**	6.4	P<0.001 (40)
5.5 versus **5**	4.9	P = 0.049 (33)
6 versus 5.5	3.4	P = 0.705 ns (14)
6 versus 4.5	1.9	P = 0.317 ns (18)
5.5 verus 4.5	1.5	P = 1.000 ns (33)
4.8 versus 5	0.7	P = 0.808 ns (34)

Note that in all cases in which the difference in sensitivity was higher than 3.4 dB, there was a significant preference for the more sensitive frequency.

**Figure 2:**
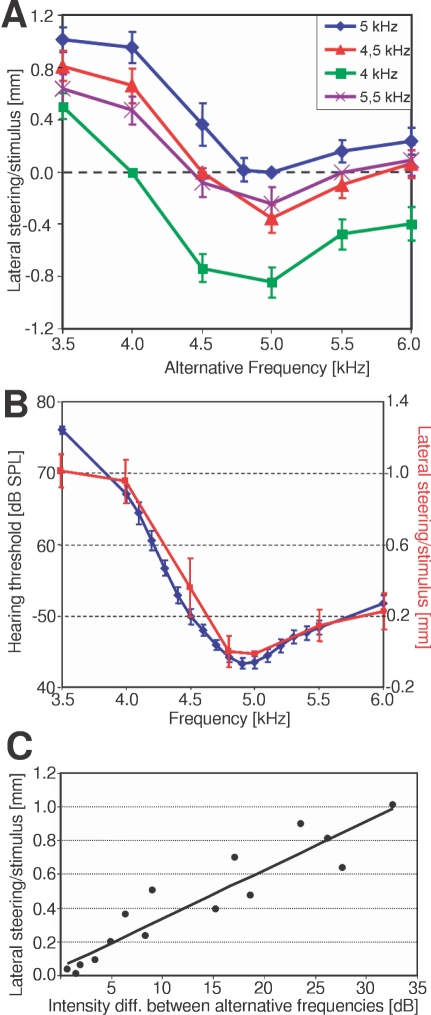
AN1-tuning is highly predictive for female choice. (A): Strength of lateral steering towards or away (positive and negative values, respectively) four calling song frequencies of 4 kHz, 4.5 kHz, 5 kHz and 5.5 kHz in a choice with calling songs ranging from 3.5 to 6 kHz. For example, 5 kHz is preferred against all CFs except 4.8 kHz, and a CF of 4 kHz is rejected in all choices except 3.5 kHz (mean±SE; N = 20). (B) The average tuning of AN1 (blue; mean±SE; N = 20) correlates strongly with the behavioural preference (lateral steering towards a CF of 5 kHz in a choice with alternative CFs from 3.5 to 6 kHz, and (C) the degree of lateral steering increases with the intensity difference due to the threshold difference in AN1 for the two alternative CFs.

We also examined the degree of preference, as evident in the quantitative value of lateral steering towards a given signal (figure 2A). The signal with a CF of 5 kHz is preferred against all other CFs in the choice paradigm, and the degree of steering increases towards a choice with lower CFs. Only between 5 kHz and 4.8 kHz there was no preference for either signal. By contrast, a signal with a CF of 4 kHz was rejected against all other CFs except in a choice with 3.5 kHz. Both, the direction and degree of preference can be fully explained by the AN1-tuning of receivers. For example, one prediction from the AN1-tuning is that all frequencies which exhibit lower thresholds compared to alternative frequencies should be preferred. In comparison with 4 kHz, all other frequencies except 3.5 kHz exhibit lower thresholds, and consequently higher afferent excitation, and are preferred against 4 kHz. Only for 3.5 kHz the threshold is about 10 dB higher compared to 4 kHz, and females steered towards 4 kHz Similarly, the average tuning of females exhibits a BF of 4.9 kHz, and thus 5 kHz activates the afferent system more strongly than any alternative CF except 4.8 kHz. The tuning of AN1 also scales quantitatively with the degree of preference. The minute difference of only 0.7 dB in a choice between 4.8 kHz and 5.0 kHz resulted in a lateral steering of only 0.03 mm, whereas a difference of 6.4 dB (in a choice 4.5 kHz and 5.0 kHz) resulted in a steering of 0.36 mm. A high threshold difference of 23.5 dB, which happens in a choice 4.0 kHz and 5.0 kHz resulted in a strong steering of 0.9 mm. The high agreement between neuronal tuning and the strength of preference is summarized in figure 2B, where the amount of steering towards the CF of 5 kHz decreases according to the decrease in threshold in AN1 between the two competing CFs. The amount of threshold difference for the two involved CFs in a choice situation (inferred from the tuning of AN1) correlates with the strength of observed steering (figure 2C), and this correlation is very strong (R^2^ = 0.87).

### A second matched filter: Peripheral directionality

Most females are tuned to a BF shifted to higher values compared to the population mean of male signals (figure 1A), which should result in selection for this trait [5]; [28]. Why did males not respond to this selection imposed by female choice, by shifting their CF to higher frequencies? Here we show that this could be due to a second matched filter in the auditory system tuned in the context of directional hearing to lower frequencies.

**Figure 3:**
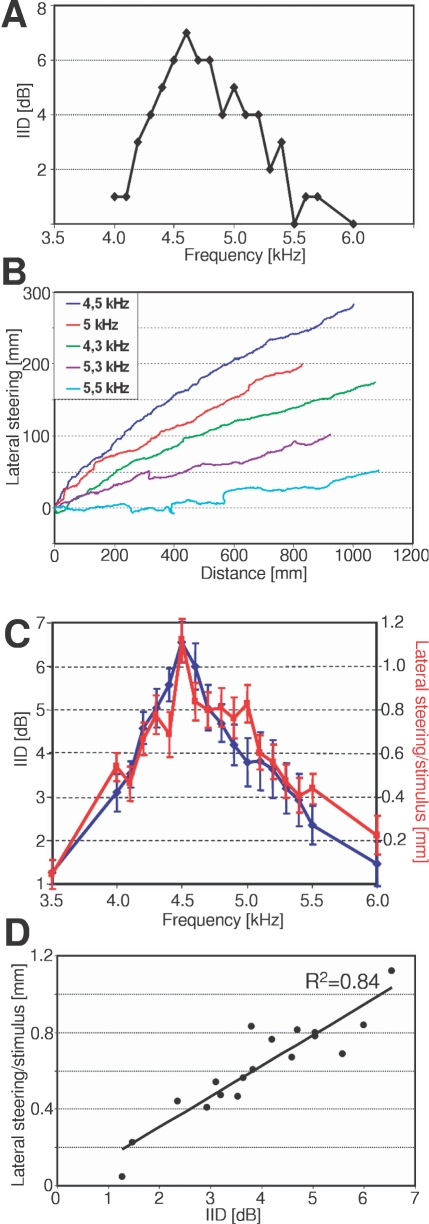
Interaural intensity differences exhibit optimum functions and determine the degree of lateral steering in no-choice paradigms. In a single female, the amount of IID provided by the peripheral directionality (A) correlates with the degree of steering (B). (C) On average, the peripheral directionality is tuned to 4.5 kHz, i.e. provides the highest IIDs (blue; mean±SE; N = 20), although optima in single females vary from 4.0 to 5.1 kHz. The lateral steering in no-choice paradigms (red; mean±SE; N = 20) also peaks at 4.5 kHz and exhibits a rather similar optimum function. The correlation of mean values between IIDs and lateral steering (n = 20) is high (D).

The average directional tuning of females is best at 4.5 kHz (figure 3A), but individual females may be directionally tuned in a frequency range from 4 kHz to 5.1 kHz. Thus, considerable variation exists in this receiver property, covering most of the range of male CFs in the population. Body size of receivers appears to be one determinant for the frequency optimum in directionality (y = −0.6x+9.41; x: body size, y: frequency optimum; R^2^ = 0.37): the smallest females provide largest IIDs around 5 kHz, whereas the largest female has its optimum at 4 kHz.

The tuning in directionality has implications for the degree of steering towards a single sound source, as evident in the directional tuning and the degree of lateral steering of an individual female (figures 3A, B). This female with a directional tuning at 4.6 kHz exhibited strongest steering in behaviour close to this maximum, but less at 5.5 kHz and 4.3 kHz, where IIDs are reduced. At a CF of 5.3 kHz, the IID of only 2 dB resulted in an even less lateral steering, and at 5.5 kHz there was no reliable steering at all, because IIDs do not exist. Notably, this female had its neuronal tuning at a BF of 5 kHz (same female as in fig. 1B), demonstrating that in the same individual the BF of AN1-frequency tuning may differ considerably from the BF of directional tuning. Figure 3C demonstrates the similarity in directional tuning and lateral steering for all females studied. The correlation between the observed lateral steering in behaviour and the amount of IIDs provided by the directional filter is very high (R^2^ = 0.84; figure 3D). On average, for all frequencies tested an increase of 1 dB in IID resulted in an increase in lateral steering of 0.15 mm.

**Figure 4:**
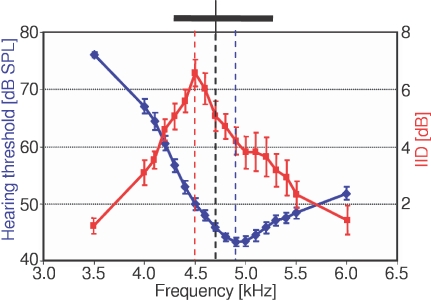
Summary of two matched filters. Whereas the average AN1-neuron is tuned to 4.9 kHz (blue), the peripheral directionality is tuned to 4.5 kHz (red). The calling song frequencies of males vary from 4.3 to 5.3 kHz (horizontal bar; mean 4.7 kHz; broken line), indicating that the combined action of both receiver biases exerts a stabilizing selection pressure on this male trait.

Thus, the frequency providing strongest stimulation for the auditory system may provide only poor directional cues. On average there is a discrepancy of 400 Hz between the two frequency optima, summarized in figure 4. These two optima are significantly different (Whitney rank sum test, p<0.001; N = 20). This is consistent with the optima seen in behaviour in the no-choice directionality test (4.5 kHz) and in the two-choice paradigm at 5.0 kHz, which are also significantly different (Whitney rank sum test, p = 0.001; N = 20). By contrast, the optimum observed in the choice situation is not different from the BF in the AN1-tuning, and the same holds true for the IID optimum and the one in the no-choice directionality test (Whitney rank sum test, p = 0.98 and 0.41, respectively; N = 20).

## Discussion

Our results are compatible with the notion that a matched filter for sound frequency exists in *G. bimaculatus*, implemented in the receptors of the receiver and read out by the pair of AN1-interneurons, which can be considered (as) the “hard-wired” preference function in this insect, since it exhibits a frequency tuning that explains about 80% of the variation in the direction and strength of phonotactic steering in a choice situation. The high correlation between the AN1 threshold differences for different CFs and the behavior of females in a choice situation indirectly confirm previous results with experimental manipulation of AN1-activity through current injection, which demonstrated that the asymmetric discharge of the pair of AN1-neurons determines the direction of positive phonotaxis [19]; [20]. Whereas the preference of *G. bimaculatus* females for a 5 kHz carrier relative to a 4.0 or 4.5 kHz carrier is consistent with the lower threshold in AN1 for 5 kHz, they appear to contradict with those of choice experiments in the sister species *G. campestris*, where a CF of 4.25 kHz was strongly preferred relative to a 5 kHz alternative [7]. Similarly, in the cricket frog the mismatch in basilar papilla tuning of females at 3.17 kHz and the mean CF of male calls at 3.69 kHz was supposed to cause mate choice for calls with lower than average CF [3]. In *G. bimaculatus* we found only one out of twenty individuals with such a low-frequency preference. However, preliminary experiments in *G. campestris* indicate that the mean AN1-tuning is at a BF of 4.4 kHz (and thus close to the preferred CF of 4.25 kHz), and the roll-off towards lower frequencies is reduced compared to *G. bimaculatus* [Kostarakos & Römer unpublished]. It remains to be tested whether in *G. campestris* the tuning in AN1 is significantly different from that in *G. bimaculatus* to explain female preference for a call parameter that conveys information concerning fitness-related traits, such as body size. However, in *G. bimaculatus* no negative correlation between CF and male body size was found [15]. Thus, when *G. bimaculatus* females reject calls of lower frequencies, they do not reject larger males.

In addition to the matched filter represented by the tuned frequency sensitivity of the AN1-neuron there exists a second matched filter in the form of the peripheral directionality of the cricket ear. The ear is a functional three-input system for sound, given by the anatomical arrangement of connecting trachea between the ears in the forelegs. In combination with the phase delay mechanism due to the medial septum this tunes the system with respect to the carrier frequency, so that for each individual reasonable IIDs are provided only in a narrow range of frequencies [16; 24]–[27]. At the population level, however, the best frequency in directional tuning can vary considerably (from 4.0 to 5.1 kHz). The tuned directionality described here (figure 3) was based on physiological measurements and confirmed previous biophysical results using laser-vibrometry [27]. However, whereas the average directionality (figures 3A, 4) would suggest a highly tuned directionality for 4.5 kHz, we found a high variation in the population of females regarding the BF of directional tuning, so that some females established largest IIDs at 4 kHz, others at 5.1 kHz. One source of this variation appears to be the body size of receivers, with largest females being directionally tuned to lower frequencies.

Sensory tuning, such as those in the neuronal tuning in AN1 and in directional tuning of the peripheral auditory system, are usually considered to impose stabilizing sexual selection on the male signal. If the best frequency of the female preference function is the same as the mean of the male CF of the calling song, sexual selection will be stabilizing, and if the best frequency differs from the mean CF, but is within the range of CFs expressed in the male population, selection can be both directional and stabilizing [10], . The latter situation is given for the neuronal tuning and directional tuning in *G. bimaculatus*. Most surprising was the fact, however, that the best frequencies in the two matched filters of female auditory pathways are not the same: The average AN1 is tuned to 4.9 kHz, whereas the directional tuning is best at 4.5 kHz. Thus, there are two different preference peaks, which may exert selection on male signals. The fact that the average CF of male calls in a population is close to 4.7 kHz (figure 4), and thus right between both receiver optima, would indicate that sexual selection is stabilizing, with both filters probably contributing to the evolution of the CF in the male signal. Recent studies in the field and under laboratory conditions indicate that female cricket *Teleogryllus commodus* exert significant multivariate stabilizing selection on call characters, one of which was the CF of the calling song [10], [32]. The existence of two frequency optima, rather than one, which influence the preference of females differing by 400 Hz may also explain at the proximate level the high phenotypic variance in this signal trait. If there was only one peak given by the tuning of AN1, this would strongly select against males calling between 4.0 and 4.5 kHz (see behavioural preference of females in figure 2A). Consider, however, a female listening to several males under field conditions, where directional information can be heavily distorted [33]–[35]. The CF of a male song close to the BF of the female's AN1-tuning will activate her afferent system strongly, but may provide little directional information (figure 4). Under these conditions it may probably not be adaptive to track and search for extensive time under non-directional cues, but rather find a less stimulating singing male on a straight path, because of reliable IIDs provided by the lower frequencies.

Why are the two involved “matched filters” mismatched to each other? First, we reject the hypothesis that the mismatch might result from a trade-off in short- versus long-range communication (for an experimental test in tungara and cricket frogs see [36]), since the CF of a cricket call does not vary with distance from the source (although higher harmonics disappear with distance). In crickets, and different from grasshoppers, the ability for hearing evolved in the context of intraspecific communication [37], [38]. If the first step in the evolution of the system happened in the context of courtship at close range, there was selection on male signals to successfully activate the auditory system of receivers, without the need for localisation, because of already existing visual contact between mates. Only later, when the advantage of air-borne sound for effective communication over long distances was used, the system had to be modified in order to produce directional cues for mate finding. In this context, various trade-offs result from the biophysics of sound production, transmission and hearing, together with the ground-living lifestyle and communication in field crickets [for a discussion of these trade-offs, see [24]. The result is a directional receiver tuned to a narrow range of frequencies (figure 4). Although our study provides only information about one source of individual variation in BF of directional tuning, with larger females being tuned to lower frequencies, variation in tuning of AN1 may be caused by different proximate factors, which may contribute to the frequency mismatch of the two “matched filters”.

Our findings also shed some light on the general discussion whether female responses to male traits based on sensory biases can be considered adaptive or not [39]–[42]. It has been argued that an important female fitness gain is the reduction in the costs of searching for mates, even if the choice does not result in offspring with superior genes for viability [40], [42]. In their discussion on the tactical design of animal signals and its importance for the evolution of signal properties the same authors listed three important aspects of the “psychological landscape of receivers”, namely the detectability, discriminability, and memorability for a given signal. Here we would add a fourth component, which we consider equally important, and difficult to obtain in the acoustic domain, namely the locatability of a signal. The importance of the filter in female directionality should, however, strongly vary with the ecological setting where choice takes place, and the higher the degradation of directional cues, the higher is the importance of large IIDs provided by the sensory system.

The high correlation between female preference and neuronal threshold values of the AN1-neuron (figure 2B) points to the importance of this neuron for mate choice decisions [19]. However, the sensory system of females does not rely on differences in relative thresholds for alternative signals, but rather these differences are translated into discharge differences of receptors and interneurons of the auditory pathway, providing females with a neuronal cue. Yet, it has to be demonstrated that such discharge differences in the pair of AN1-neurons correlate with the behavior of females as well. Current experiments with simultaneous bilateral recordings of this pair of neurons aim to demonstrate this. Preliminary results suggest that on average there is also a high correlation between the bilateral discharge differences in the pair of AN1-neurons and the steering responses observed in the choice paradigm and directionality, even at the high intensity of 80 dB SPL used in the behavioral experiments.

## Materials and Methods

Female crickets (*Gryllus bimaculatus* de Geer) were obtained from a local supplier as last instars and raised individually to adulthood to maintain phonotactic responsiveness. They were used for behavioural experiments starting one week after the final moult. Phonotactic behaviour was studied using a highly sensitive trackball system which allowed measuring the walking behaviour of tethered females (for details of the system see [43], [44]. The advantage of this set-up is that while females moved the trackball system with their legs, their body position relative to the speakers, and thus the acoustic conditions for the ears, remained constant throughout the phonotactic path. An optical mouse sensor (Logitech, MX518) positioned at the south pole of the trackball recorded the movements of the trackball in the forward-backward x-axis, and lateral left-right y-axis on two separate channels. The trackball data were sampled online at 10 kHz, controlled by custom-programmed software.

### Acoustic stimulation

Sound stimuli were generated with Cool Edit software and broadcast by standard PC audio boards, a stereo power amplifier (NAD 214) and two mid-range speakers (Tonsil GTC 10/60). Speakers were placed at a distance of 50 cm and an angle of 30° to the right and 30° left of the longitudinal body axis of the female. Sound pressure levels were calibrated at the position of the female to 80 dB SPL using a sound level meter (Rion NL-21) and integrated microphone (UC-52), and are given in SPL relative 20 µPa. The standard stimulus had a carrier frequency of 5.0 kHz, a pulse duration of 23 ms, an interpulse interval of 16 ms, with four pulses per chirp. Four chirps with an interchirp interval of 230 ms were followed by a silent interval of 750 ms before this series of chirps was repeated in an endless loop. This temporal pattern was used for all stimuli which varied in CF from 3.5 to 6 kHz, in increments of 0.5 kHz or 0.1 kHz. In female choice experiments with different CFs the temporal pattern was presented via the opposite speakers in a time-shifted fashion, so that one speaker presented the first chirp of the stimulus, and the other speaker the alternative chirp during the interchirp interval of the opposite stimulus. In the standard female choice paradigm frequencies of 3.5; 4; 4.5; 5; 5.5 and 6 kHz were used and its effect on the preference of females compared with each of the other frequencies. For frequencies of 4; 4.5; 5 and 5.5 kHz we also performed the control experiment, by stimulating females with the same choice, but switched frequencies between speakers. These controls are important to determine the degree of asymmetry, and the mean values for the lateral steering represents a reliable value for the preference for a specific frequency.

From the data of the optical mouse sensor coding for the lateral movement of the insect we calculated the actual lateral steering by which the female steered to either side, and the lateral deviation of the animal from a straight path. The steering to one of two alternative stimuli indicates the preference for a specific frequency compared to the alternative frequency. Lateral steering was calculated for the time of 370 ms, i.e. for the presentation of the chirp and the following interchirp interval, which allowed also analysing the steering to the alternative chirp in choice experiments, which occurred in the interchirp interval of the other sound channel. A behavioural latency of 80 ms was considered for the analysis, since the onset of lateral steering in walking and flying crickets is delayed by this time relative to the stimulus onset [42].

Phonotactic tracks were recorded for one minute, so that the average lateral steering towards 120 chirps could be calculated. For each millisecond, a running average of the next 10 ms was calculated to achieve a higher linearity. Positive and negative values indicated a steering to the right or left speaker, respectively.

### Neurophysiology

Extracellular recordings of the discharges of a prominent interneuron, the AN1-neuron, were used to examine both the tuning of the hearing system and the peripheral directionality. Action potential activity was recorded with extracellular tungsten hook-electrodes placed at the connectives between prothoracic and suboesophageal ganglia. Insects were shortly anesthesized with chlorethylene and fixed ventral side up on a thin (1 mm) platform with dental wax. The forelegs were fixed in the natural walking position onto thin wires (diameter 0.6 mm). The preparation was sealed with petroleum jelly to prevent desiccation of the connectives. AN1-discharges were amplified by a custom-made amplifier, visualized on an oscilloscope (Agilent 54616B) and monitored through headphones. The threshold for each stimulus was defined as the SPL which elicited at least one AP in each syllable in at least three out of five stimulations.

Neurophysiological experiments were performed in an acoustically isolated Faraday-cage at room temperature between 21–23°C. A speaker (Raveland MHX 138) was placed at a distance of 40 cm, at an angular separation of 30° left or 30° right of the longitudinal body axis. Model songs identical to those used in behaviour were generated, amplified and attenuated in steps of 1 dB using a Tucker-Davis system (Alachua, Florida). The tuning of AN1 was determined with ipsilateral stimulation at frequencies ranging from 3.5 to 6 kHz, in increments of 100 or 500 Hz. In order to measure only the directionality provided by the anatomical arrangement of the acoustic tracheae in the periphery, inhibitory central nervous interactions were eliminated by cutting the contralateral leg nerve with the afferent auditory fibres of the opposite ear. IIDs were calculated by measuring the thresholds of the AN1-neuron for frequencies between 3.5 to 6 kHz with ipsilateral and contralateral stimulation at an angular frontal deviation of 30°. The threshold differences between ipsi- and contralateral stimulation represent the IID for a given frequency at a stimulation angle of ±30°, similar to biophysical measurements using laser-vibrometry [27]). Pronotum width was measured as an index of structural body size to the nearest 0.1 mm, using a digital calliper.

## References

[1] Capranica RR, Moffat AJM, Ewert JP, Capranica RR, Ingle D (1983). Neurobehavioral correlates of sound communication in anurans.. Vertebrate Neuroethology.

[2] Gerhardt HC, Schwarz JJ, Ryan MJ (2001). Auditory tuning and frequency preferences in anurans.. Anuran communication.

[3] Wilczynski W, Keddy-Hector A, Ryan MJ (1992). Call patterns and basilar papilla tuning in cricket frogs. I. Differences among populations and between sexes.. Brain Behav Evol.

[4] Witte K, Farris HE, Ryan MJ, Wilczynski W (2005). How cricket frog females deal with a noisy world: habitat-related differences in auditory tuning.. Behav Ecol.

[5] Gerhardt HC, Huber F (2002). Acoustic communication in insects and anurans..

[6] Jennions MD, Petrie M (1997). Variation in mate choice and mating preferences: a review of causes and consequences.. Biol. Rev..

[7] Simmons LW, Ritchie MG (1996). Symmetry in the songs of crickets.. Proc. R. Soc. Lond. B.

[8] Simmons LW, Zuk M, Rotenberry JT (2001). Geographic variation in female preference functions and male songs of the field cricket *Teleogryllus oceanicus*.. Evolution.

[9] Scheuber H, Jacot A, Brinkhof MWG (2003). The effect of past condition on a multicomponent signal.. Proc R Soc Lond B.

[10] Bentsen CL, Hunt J, Jennions MD, Brooks R (2006). Complex multivariate sexual selection on male acoustic signaling in a wild population of *Teleogryllus commodus*.. Am Nat.

[11] Scheuber H, Jacot A, Brinkhof MWG (2003). Condition dependence of a multicomponent sexual signal in the field cricket *Gryllus campestris*.. Anim Behav.

[12] Nocke H (1972). Physiological aspects of sound communication of crickets (*Gryllus campestris* L.).. J Comp Physiol A.

[13] Helversen von D, Balakrishnan R, Helversen von O (2004). Acoustic communication in a duetting grasshopper: receiver response variability, male strategies and signal design.. Anim Behav.

[14] Bennet-Clark HC (1998). Size and scale effects as constraints in insect sound communication.. Phil Trans R Soc Lond B.

[15] Ferreira M, Ferguson JWH (2002). Geographic variation in the calling song of the field cricket *Gryllus bimaculatus* (Orthoptera: Gryllidae) and its relevance to mate recognition and mate choice.. J Zool Lond.

[16] Hill KG, Boyan GS (1976). Directional hearing in crickets.. Nature.

[17] Hunt J, Brooks R, Jennions MD (2005). Female mate choice as a condition-dependent life-history trait.. Am Nat.

[18] Verburgt L, Fergusen JWH, Weber T (2008). Phonotactic response of female crickets on the Kramer treadmill: methodology, sensory and behavioural implications.. J Comp Physiol A.

[19] Schildberger K, Hörner M (1988). The function of auditory neurons in cricket phonotaxis. I. Influence of hyperpolarization of identified neurons on sound localization.. J Comp Physiol A.

[20] Atkins G, Henley J, Handysides R, Stout J (1992). Evaluation of the behavioral roles of ascending auditory interneurons in calling song phonotaxis by the female cricket *(Acheta domesticus)*. J Comp Physiol A.

[21] Huber F, Kleindienst H-U, Weber T, Thorsen J (1984). Auditory behaviour of the cricket III. Tracking of male calling song by surgically and developmentally one-eared females and the curious role of the anterior tympanum.. J Comp Physiol A.

[22] Pollack GS (1986). Discrimination of calling song models by the cricket, *Teleogryllus oceanicus*: The influence of sound direction on neural coding of the stimulus temporal pattern and on phonotactic behavior.. J Comp Physiol A.

[23] Schildberger K (1994). The auditory pathway of crickets: Adaptations for intraspecific communication. In: Neural Basis of Behavioural Adaptations. Schildberger K, Elsner N. editors.. Fortschr Zool Stuttgart, New York, Gustav Fischer.

[24] Michelsen A, Hoy RR, Popper AN, Fay RR (1998). Biophysical basis of sound localization in insects.. Comparative hearing: Insects.

[25] Thorsen J, Weber T, Huber F (1982). Auditory behaviour of the cricket. II. Simplicity of calling-song recognition in Gryllus and anomalous phonotaxis at abnormal carrier frequencies.. J Comp Physiol A.

[26] Wendler G, Löhe G (1993). The role of the medial septum in the acoustic trachea of the cricket *Gryllus bimaculatus*.. J Comp Physiol A.

[27] Michelsen A, Löhe G (1995). Tuned directionality in cricket ears.. Nature.

[28] Ryan MJ, Keddy-Hector A (1992). Directional patterns of female mate choice and the role of sensory biases.. Am Nat.

[29] Ryan MJ, Rand AS (1993). Sexual selection and signal evolution: the ghost of biases past.. Phil Trans R Soc Lond B.

[30] Gerhardt HC (1991). Female mate choice in treefrogs: static and dynamic acoustic choice criteria.. Anim Behav.

[31] Greenfield MD (2002). Signallers and receivers: mechanisms and evolution of arthropod communication..

[32] Brooks R, Hunt J, Blows MW, Smith MJ, Bussière LF, Jennions MD (2005). Experimental evidence for multivariate stabilizing sexual selection.. Evolution.

[33] Rheinlaender J, Römer H (1986). Insect hearing in the field I. The use of identified nerve cells as “biological microphones”.. J Comp Physiol A.

[34] Michelsen A, Rohrseitz K (1997). Directional sound processing and interaural sound transmission in a small and a large grasshopper.. J Exp Biol.

[35] Gilbert F, Elsner N (2000). Directional hearing of a grasshopper in the field.. J Exp Biol.

[36] Sun LX, Wilczynski W, Rand AS, Ryan MJ (2001). Trade-off in short- and long-distance communication in túngara (*Physalaemus pustulosus*) and cricket (*Acris crepitans*) frogs.. Behav Ecol.

[37] Sharov AG (1968). Phylogeny of the Orthopteroidea (Transl 1971, Israel Program for Scientific Translation, Jerusalem).. Trans Paleontol Inst Acas Sci *USSR*.

[38] Dirsh VM (1975). A preliminary revision of the families and sub-families of Acridoidea (Orthoptera, Insecta).. Bull Brit Mus (Nat hist).

[39] Kirkpatrick M, Bradbury JW, Andersson MB (1987). The evolutionary forces acting on female mating preferences in polygynous animals.. Sexual selection: testing the alternatives.

[40] Guilford T, Dawkins MS (1991). Receiver psychology and the evolution of animal signals.. Anim Behav.

[41] Hill GE (1994). Geographic variation in male ornamentation and female preference in the house finch: a comparative test of models of sexual selection.. Behav Ecol.

[42] Dawkins MS, Guilford T (1996). Sensory bias and the adaptiveness of female choice.. Am Nat.

[43] Hedwig B, Poulet JFA (2004). Complex auditory behaviour emerges from simple reactive steering.. Nature.

[44] Hedwig B, Poulet JFA (2005). Mechanisms underlying phonotactic steering in the cricket *Gryllus bimaculatus* revealed with a fast trackball system.. J Exp Biol.

